# A FDG-PET radiomics signature detects esophageal squamous cell carcinoma patients who do not benefit from chemoradiation

**DOI:** 10.1038/s41598-020-74701-w

**Published:** 2020-10-19

**Authors:** Yimin Li, Marcus Beck, Tom Päßler, Chen Lili, Wu Hua, Ha Dong Mai, Holger Amthauer, Matthias Biebl, Peter C. Thuss-Patience, Jasmin Berger, Carmen Stromberger, Ingeborg Tinhofer, Jochen Kruppa, Volker Budach, Frank Hofheinz, Qin Lin, Sebastian Zschaeck

**Affiliations:** 1 Department of Radiation Oncology, First Affiliated Hospital of Xiamen University, No. 55 Zhenhai Road, Siming District, Xiamen City, 361003 Fujian Province China; 2 Department of Radiation Oncology, University of California Davis School of Medicine, Sacramento, CA USA; 3 Charité – Universitätsmedizin Berlin, Corporate Member of Freie Universität Berlin, Humboldt-Universität zu Berlin, and Berlin Institute of Health, Department of Radiation Oncology, Augustenburger Platz 1, 13353 Berlin, Germany; 4 Department of Nuclear Medicine, The Xiamen First Affiliated Hospital of Xiamen University, Xiamen, People’s Republic of China; 5 Department of Surgery, Campus Charité Mitte and Campus Virchow Klinikum, Charité, Universitätsmedizin Berlin, Berlin, Germany; 6 Department of Hematology, Oncology and Tumor Immunology, Charité - Universitätsmedizin Berlin, Campus Virchow-Klinikum, Berlin, Germany; 7 German Cancer Research Center (DKFZ), Heidelberg, and German Cancer Consortium (DKTK) Partner Site Berlin, Berlin, Germany; 8 Department of Biostatistics and Clinical Epidemiology, Charité - Universitätsmedizin Berlin, Berlin, Germany; 9 Helmholtz-Zentrum Dresden-Rossendorf, PET Center, Institute of Radiopharmaceutical Cancer Research, Dresden, Germany; 10 Berlin Institute of Health (BIH), Anna-Louisa-Karsch 2, 10178 Berlin, Germany

**Keywords:** Prognostic markers, Oesophageal diseases, Tumour biomarkers, Oesophageal cancer, Cancer imaging

## Abstract

Detection of patients with esophageal squamous cell carcinoma (ESCC) who do not benefit from standard chemoradiation (CRT) is an important medical need. Radiomics using 18-fluorodeoxyglucose (FDG) positron emission tomography (PET) is a promising approach. In this retrospective study of 184 patients with locally advanced ESCC. 152 patients from one center were grouped into a training cohort (n = 100) and an internal validation cohort (n = 52). External validation was performed with 32 patients treated at a second center. Primary endpoint was disease-free survival (DFS), secondary endpoints were overall survival (OS) and local control (LC). FDG-PET radiomics features were selected by Lasso-Cox regression analyses and a separate radiomics signature was calculated for each endpoint. In the training cohort radiomics signatures containing up to four PET derived features were able to identify non-responders in regard of all endpoints (DFS p < 0.001, LC p = 0.003, OS p = 0.001). After successful internal validation of the cutoff values generated by the training cohort for DFS (p = 0.025) and OS (p = 0.002), external validation using these cutoffs was successful for DFS (p = 0.002) but not for the other investigated endpoints. These results suggest that pre-treatment FDG-PET features may be useful to detect patients who do not respond to CRT and could benefit from alternative treatment.

## Introduction

Esophageal squamous cell carcinoma (ESCC) is a tumor with an unfavorable outcome and a high global disease burden, especially in Asia and Southern Africa. Established treatment options are surgery alone (for limited stages of disease), preoperative chemoradiation (CRT) followed by surgery, or definitive CRT. Several phase-III studies addressed the role of preoperative CRT compared to surgery alone in locally advanced ESCC and reported an improvement of disease-free survival (DFS) and overall survival (OS) by the additional use of CRT^1–3^. Despite improved treatment results, up to 20% of patients do not benefit from preoperative CRT, as tumors of these patients do not present regressive changes at the time of surgery^2^. These tumors may progress despite CRT or develop distant metastases during fractionated CRT^4^. While for the majority of patients CRT might still provide palliative relief, these patients obviously would need a different treatment approach. Future aims to personalize treatment should enable clinicians to detect these highly chemo-radioresistant tumors already prior to the start of preoperative CRT and potentially treat these patients with alternative treatment regimes.

Radiomics, i.e. generation of quantifiable parameters of certain imaging features, might be a useful tool for patient stratification and treatment individualization. Radiomics can be performed on routinely acquired clinical imaging data, e.g. from ^18^F-fluorodeoxyglucose (FDG) positron emission tomography (PET), which is often used for staging and radiation treatment planning of ESCC patients. Radiomics refers to the analysis of textural and other features that contain parameters which are not amenable to the human eye or conventional assessment (e.g. routinely used PET parameters like standardized uptake values). Therefore, FDG-PET radiomics might be a promising approach to decipher specific tumor phenotypes that are associated with CRT responsiveness or resistance. One important drawback of this approach is the widespread use of single institutional data for the establishment and validation of radiomics signatures. Due to the plethora of parameters and the corresponding risk of statistical overfitting, independent external validation is an important prerequisite for the further implementation of radiomics signatures into clinical practice. In the present study we developed a radiomics risk score using the pre-treatment PET imaging data of Chinese patients. This score was subsequently validated using an internal patient cohort, and additionally an external cohort of European patients. Primary endpoint for this study was disease-free survival (DFS).

## Results

**Figure 1:**
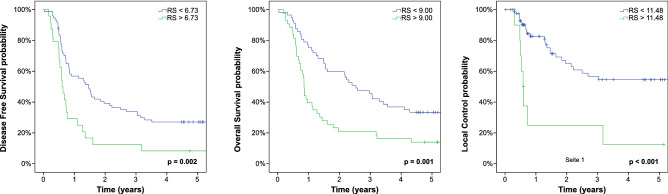
Training cohort. Kaplan–Meier estimates with prognostic groups split by endpoint-specific radiomics signatures (RS) into high and low-risk population.

Prognostic radiomics signatures could be established in the training cohort for the endpoints DFS, OS and local control (LC). Calculation of a freedom from distant metastases (FFDM) signature was not successful due to the low number of events within the training cohort. The obtained radiomics signatures showed a high prognostic impact and a significant discrimination of high and low-risk patients over a broad range of cutoff values. Details on the calculation of the radiomics signatures can be found in Supplementary Table S2. Independence from known clinical risk factors was assessed by multivariate analysis of the radiomics signature and all potentially relevant clinical parameters. Additionally no correlation with other clinical or treatment parameters was observed (Supplementary Table S3). Maximal standardized uptake values (SUV_max_) did not show a high prognostic value in this cohort of patients, as already published^5^. Including radiomics signatures as metric parameters in the model revealed their significant interaction with DFS, LC and OS (p-values < 0.001, 0.003 and 0.001 respectively). The radiomics signatures showed a high correlation with metabolic tumor volume (MTV) (Spearman coefficient: DFS signature r = 0.83, p < 0.001; LC signature r = 0.96, p < 0.001; OS signature r = 0.86, p < 0.001), therefore MTV was not included in multivariate analyses. In contrast to the radiomics signatures it was not possible to identify very-high risk patients by the use of MTV only, except for LC (Supplementary Figs. S1–S3 for an example of the best high-risk discriminations). Additionally SUVmax was not able to identify high-risk groups (Supplementary Figs. S4–S6). Upon multivariate testing the radiomics signatures remained an independent prognostic factor for DFS, OS and LC. Table 1 summarizes the results of uni- and multivariate analyses for all endpoints of the training cohort. Figure 1 shows the Kaplan–Meier estimates for all endpoints with radiomics signatures developed to detect patients with very unfavorable outcome. By this approach a significant discrimination of risk-groups was feasible for all endpoints (DFS: cutoff for radiomics signature = 6.73, p = 0.002; OS: cutoff for radiomics signature = 9.00, p = 0.001; and LC: cutoff for radiomics signature = 11.48, p < 0.001).

**Table 1:** Training cohort.

Parameter	Univariate HR (range)	Univariate p	Multivariate HR (range)	Multivariate p
**Disease free survival**				
Age	1.01 (0.99–1.03)	0.47		
Gender	1.09 (0.65–1.83)	0.75		
Grading	0.81 (0.53–1.23)	0.32		
UICC group	1.01 (0.76–1.33)	0.95		
Type of chemotherapy	0.87 (0.64–1.18)	0.37		
Radiation dose	0.96 (0.91–1.01)	0.101		
MTV	1.00 (1.00–1.00)	** < 0.001**		
SUV_max_	1.00 (1.00–1.00)	0.068		
Radiomics signature	8.64 (2.7–27.1)	** < 0.001**		
**Local control**				
Age	0.99 (0.96–1.03)	0.74		
Gender	1.14 (0.53–2.43)	0.74		
Grading	0.75 (0.39–1.41)	0.37		
UICC group	0.80 (0.56–1.16)	0.24		
Type of chemotherapy	1.11 (0.72–1.73)	0.63		
Radiation dose	1.00 (0.92–1.09)	0.99		
MTV	1.00 (1.00–1.00)	**0.004**		
SUV_max_	1.00 (1.00–1.00)	0.55		
Radiomics signature	1.19 (1.06–1.34)	**0.003**		
**Overall survival**				
Age	1.01 (0.99–1.04)	0.26		
Gender	1.19 (0.71–2.01)	0.50		
Grading	0.73 (0.47–1.12)	0.15		
UICC group	1.09 (0.82–1.46)	0.54		
Type of chemotherapy	0.80 (0.59–1.08)	0.14		
Radiation dose	0.95 (0.90–0.99)	**0.035**	0.94 (0.89–0.99)	**0.02**
SUV_max_	1.00 (1.00–1.00)	**0.073**		
MTV	1.00 (1.00–1.00)	**0.001**		
Radiomics signature	6.93 (2.28–21.05)	**0.001**	7.47 (2.43–22.98)	** < 0.001**

Univariate and multivariate cox regression analyses of clinical parameters, treatment characteristics, the conventional PET parameter metabolic tumor volume (MTV) and radiomics signatures with respect to DFS, LC and OS. Due to the high correlation of radiomics signatures and MTV only radiomic signatures were included in multivariate analysis.

**Figure 2:**
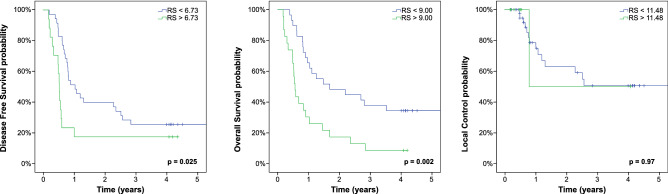
Internal validation cohort. Kaplan–Meier estimates with prognostic groups split by endpoint-specific radiomics signatures (RS) into high and low-risk population.

In a second step the radiomics signatures were calculated for the internal validation cohort. We were able to validate the prognostic value with the generated cutoff values for DFS (p = 0.025) and OS (p = 0.002) but not LC (p = 0.97). Additionally the MTV cutoff values of the training cohort also failed to identify patients with high risk for local recurrence in the internal validation cohort (Supplementary Fig. S7). Table 2 shows the hazard ratios for all investigated endpoints with non-binarized radiomics signatures. Figure 2 shows the corresponding Kaplan–Meier estimates with the cutoff values generated in the training cohort. To account for survival imbalances between training cohort and internal validation cohort that might have affected the LC endpoint, synthetic minority oversampling technique (SMOTE) was used to balance the data. Even when using SMOTE, it was not possible to validate the LC prognostic signature in this cohort (Supplementary Fig. S8).

**Table 2:** Internal validation cohort.

Parameter	Univariate HR (range)	Univariate p	Multivariate HR (range)	Multivariate p
**Disease free survival**				
Age	1.07 (1.01–1.13)	**0.014**	0.99 (0.95–1.02)	0.375
Gender	0.65 (0.24–1.77)	0.40		
Grading	1.06 (0.63–1.78)	0.84		
UICC group	0.84 (0.42–1.66)	0.51		
Type of chemotherapy	0.96 (0.65–1.40)	0.81		
Radiation dose	0.94 (0.87–1.02)	0.16		
SUV_max_	1.00 (1.00–1.00)	0.55		
MTV	1.00 (1.00–1.00)	** < 0.001**		
Radiomics signature	10.18 (2.37–43.80)	**0.002**	9.74 (2.17–43.71)	**0.003**
**Local control**				
Age	0.94 (0.83–1.06)	0.32		
Gender	1.38 (0.12–15.50)	0.80		
Grading	1.29 (0.57–2.94)	0.54		
UICC group	0.001–15.58	0.39		
Type of chemotherapy	1.47 (0.79–2.74)	0.22		
Radiation dose	0.94 (0.81–1.09)	0.41		
SUV_max_	1.00 (1.00–1.00)	0.29		
MTV	1.00 (1.00–1.00)	0.44		
Radiomics signature	1.03 (0.85–1.25)	0.74		
**Overall survival**				
Age	1.08 (1.01–1.16)	**0.024**	0.98 (0.95–1.02)	0.323
Gender	0.34 (0.08–1.35)	0.11		
Grading	0.97 (0.58–1.65)	0.92		
UICC group	0.63 (0.22–1.82)	0.59		
Type of chemotherapy	0.88 (0.60–1.29)	0.52		
Radiation dose	0.93 (0.86–1.02)	0.11		
SUV_max_	1.00 (1.00–1.00)	0.76		
MTV	1.00 (1.00–1.00)	** < 0.001**		
Radiomics signature	15.77 (2.98–83.48)	**0.001**	15.63 (2.87–85.21)	**0.001**

Univariate and multivariate cox regression analyses of clinical parameters, treatment characteristics, the conventional PET parameter metabolic tumor volume (MTV) and radiomics signatures with respect to DFS, LC and OS. Due to the high correlation of radiomics signatures and MTV, only radiomic signatures were included in case of multivariate testing.

**Figure 3:**
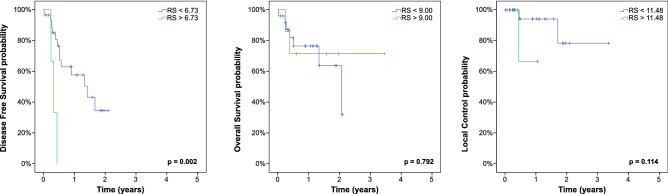
External validation cohort. Kaplan–Meier estimates with prognostic groups split by endpoint-specific radiomics signatures (RS) into high and low-risk population.

After successful internal validation, external validation was performed in a completely independent group of patients. Despite several differences regarding patient characteristics and treatment, it was possible to validate the radiomics signature cutoff for DFS (p = 0.002) but not for OS (p = 0.792) or LC (p = 0.114), the corresponding Kaplan–Meier plots are shown in Fig. 3. The non-binarized radiomics signatures did not show an association with DFS, OS or LC, see Table 3 for a summary of the univariate cox regression analyses. The MTV cutoff of the training cohort did not discriminate LC risk groups in this independent cohort (Supplementary Fig. S9).

**Table 3:** External validation cohort.

Parameter	Univariate HR (range)	Univariate p
**Disease free survival**		
Age	1.07 (1.01–1.13)	**0.014**
Gender	0.65 (0.24–1.77)	0.40
Grading	0.96 (0.28–3.25)	0.94
UICC group	0.001–12.74	0.99
Type of chemotherapy	1.29 (0.99–1.70)	0.06
Radiation dose	1.03 (0.97–1.09)	0.35
SUV_max_	1.00 (1.00–1.00)	0.39
MTV	1.00 (1.00–1.00)	0.70
Radiomics signature	1.03 (0.33–3.22)	0.95
**Local control**		
Age	0.94 (0.83–1.06)	0.32
Gender	1.38 (0.12–15.50)	0.80
Grading	0.35 (0.27–38.57)	0.33
UICC group	0.001–15.58	0.73
Type of chemotherapy	1.73 (0.95–3.17)	**0.034**
Radiation dose	1.03 (0.90–1.18)	0.72
SUV_max_	1.00 (1.00–1.00)	0.34
MTV	1.00 (1.00–1.00)	0.43
Radiomics signature	1.20 (0.88–1.64)	0.21
**Overall survival**		
Age	1.08 (1.01–1.16)	**0.024**
Gender	0.34 (0.08–1.35)	0.11
Grading	0.82 (0.16–4.26)	0.82
UICC group	0.001–13.02	0.99
Type of chemotherapy	1.47 (1.04–2.09)	**0.02**
Radiation dose	1.10 (0.98–1.22)	0.08
SUV_max_	1.00 (1.00–1.00)	0.85
MTV	1.00 (1.00–1.00)	0.93
Radiomics signature	0.85 (0.28–2.63)	0.78

Univariate cox regression analyses of clinical parameters, treatment characteristics, the conventional PET parameter metabolic tumor volume (MTV) and radiomics signatures with respect to DFS, LC and OS.

## Discussion

Identification of patients who do not benefit from current therapeutic approaches is a pivotal challenge. This holds especially true for locally advanced ESCC since all patients receive CRT. CRT is either applied preoperatively which has been shown to be superior to surgery alone in several randomized trials or as definitive CRT for organ preservation^2,3^. Two randomized phase-III studies compared definitive CRT and preoperative CRT followed by surgery and did not find a statistical significant OS benefit in favor of the additional surgery but an increased number of loco-regional recurrences in the organ preservation arm^6,7^. After preoperative CRT tumor specimens of up to 20% of patients do not show major signs of regression^2^, these patients would be candidates for alternative treatment approaches that might comprise immediate surgery, checkpoint inhibition, image based radiation dose escalation or other^8–12^.

Here we were able to demonstrate that a pre-therapeutic radiomics signature is able to identify patients at risk for early tumor recurrence or death. The prognostic value was externally validated when using a pre-specified cutoff value. This is a remarkable finding since patients´ characteristics and treatment between participating centers differed considerably. However the prognostic value for the calculated signature could not be validated by non-dichotomized cox-regression. In addition the radiomics signatures failed in the further investigated endpoints (OS and LC). The inability to validate all signatures might be due to small group sizes (especially in the external validation cohort) and a relatively large number of intercurrent deaths within the internal validation cohort hampering validation of the LC signature. Nonetheless it is remarkable that it was feasible to confirm a radiomics signature for DFS.

One important drawback of current radiomics strategies is the variability of tumor delineation. This holds true for any diagnostic imaging data but especially for computed tomography (CT) since demarcation to non-tumor affected esophagus can be challenging. Recently, it has been shown that inter-observer delineation variability influences radiomics parameters significantly and that semi-automated segmentation algorithms are more robust regarding feature extraction^13–15^. One big advantage of PET imaging is the high signal intensity of the standard PET tracer FDG in malignant tissue. This enables automatic or semi-automatic delineation of tumor volumes that are much less prone to inter-observer variability and therefore lead to a better reproducibility^16,17^. Nonetheless a major drawback of PET images is the poor spatial resolution and cross-fade effects on neighboring voxels. Given the limitations of PET imaging the use of radiomics on PET data is controversial^18^. A recent analysis of a large cohort of patients with esophageal cancer was not able to validate the prognostic value of pre-therapeutic quantitative PET imaging features^19^. However Re-Staging PET seems to deliver valuable prognostic information, both by radiomics approaches^20^ but also by conventional analyses of tumor metabolism^21,22^. Nevertheless for potential non-responders to CRT it would be pivotal to identify these patients prior to therapy. The prognostic model may be further improved by the implementation of CT derived parameters, as a recent multicenter study showed promising results regarding risk stratification based on CT data^23^.

Another important prerequisite for the clinical application of radiomics features is robustness of features to image processing steps. Whybra and colleagues investigated the stability of PET features to interpolation. All PET features identified in our study were categorized as either stable or correctable, with all features except GLCM_IDN classified as very stable (correlation coefficients of 0.9)^24^.

One strength of our study is the large cohort of patients and the internal and external validation of the radiomics signature. An important limitation regards the differences in patient characteristics: Chinese patients were all treated with definitive CRT, while European patients were treated with definitive or preoperative CRT. However the vast majority of European patients was treated with definitive CRT. Another limitation regards the imbalance between the internal exploration and validation cohort. When designing this study we decided to allocate patients randomly to the exploration or internal validation cohort and calculate the optimal radiomic signatures in all patients of the exploration cohort. This may be a reason why internal validation of the local control signature was not successful. Another weakness is the relatively short follow-up time in the European cohort of patients leading to few events for each endpoint (OS, LC), limiting the external validation to DFS with a higher number of events. The relatively short follow-up might be justified by the aim of the study: Identification of patients with dismal outcome defined by tumor persistence, early recurrence or risk death. The heterogenous treatment might however affect other endpoints, especially LC. Another shortcoming is the strong correlation of selected radiomics parameters and the derived radiomics signatures with the standard PET parameter MTV. As comprehensively discussed in a current opinion paper by Buvat and Orlhac, this is a common and well-known limitation of several PET based radiomcis approaches^25^. Nonetheless the important clinical question in our study was the identification of non-responders to CRT. At least in the training cohort MTV did not show convincing results regarding this specific aim, except for LC. In other words MTV is highly prognostic but lacks a high sensitivity do detect non-responders since also very large tumors can potentially be highly CRT sensitive. Supplementary Fig. S10 shows examples of two high risk patients with nearly the same DFS radiomics risk signature but large differences in MTV. We think the identified radiomics signature has potential to detect non-responders with a relatively high sensitivity (2 year EFS rates of 11%, 18% and 0% in each individual cohort of patients). One important limitation of our approach is the use of non-isotropic voxels as some textural features might benefit from re-sampling to isotropic voxels^26^. Other limitations of our study include the use of the fixed-bin intensity discretization only, instead of implementing both most commonly used approaches and differences in PET scanners and acquisition protocols and consecutive reconstruction algorithms. Several methods like ComBat harmonization can potentially be used to account for these differences and have shown improved external model validation in radiomics studies^27,28^. Variance analysis of the radiomic parameters and standard PET parameters showed significant differences between the two independent cohorts (Supplementary Table S6), very likely due to differences during clinical image aquisition. Nonetheless, harmonization using this method would be hardly possible in an interventional trial when patients are enrolled consecutively and treatment decision has to be done immediate after imaging. Therefore, we decided not to include these methods to see how our model performs in a routine clinical practice scenario.

Taken together our study shows that a radiomics signature might be a clinically useful tool for detection of ESCC patients with dismal outcome after current CRT based standard treatment approaches. However it is not possible to further discriminate if this is due to radioresistance or resistance to the concomitant chemotherapy, since patients received combined treatment. Additionally the external validation cohort was relatively small, therefore further prospective validation in a larger cohort of patients is warranted to use this signature for future aims on treatment individualization.

## Methods

### Inclusion criteria

Inclusion criteria for this retrospective study were:

- Histologically confirmed ESCC treated with normo-fractionated CRT, curative intent and prescribed radiation doses in case of definitive CRT between 50 and 66 Gy. Or (external validation cohort only) preoperative CRT with 41.4 Gy.
- Staging FDG-PET performed before any radiotherapy or chemotherapy

### Patients and treatment

In total, 184 patients were included in this study. A summary of patient and tumor characteristics is given in Supplementary Table S1.

Pseudonymisation was performed and consecutive numbers were randomly assigned to all patients starting with the Chinese cohort of patients treated at the University hospital Xiamen. Patients with the numbers 1 to 100 (n = 100) were used as training data set and patients with the subsequent numbers 101 to 152 (n = 52) were used for internal validation of the radiomics model. For subsequent external validation, 32 patients treated at the Charité were used (patients 153 to 184).

152 patients from the Department of Radiation Oncology of the University Hospital Xiamen were included in this study. Imaging and treatment of patients have been previously described^29,30^. Briefly, patients received definitive CRT between 2009 and 2013, mostly using intensity modulated radiotherapy (IMRT). All patients received a radiation dose of 50 Gy to the tumor, affected and elective lymph nodes and safety margins. Thereafter a consecutive boost of 4–16 Gy was prescribed to tumor/affected lymph nodes (average total dose: 57 Gy) with reduced margins. Concomitant chemotherapy consisted of two cycles of cisplatin (25 mg/m^2^/day, days 1–3 and days 29–31) and either paclitaxel (135 mg/m^2^/day, day 1 and day 29) or 5-fluorouracil (500 mg/m^2^/day, days 1–5 and days 29–33).

The external validation cohort consisted of 32 patients with pre-therapeutic FDG-PET scans available. 22 patients received definitive CRT (50–66 Gy) while 10 patients received preoperative CRT to a total dose of 41.4 Gy followed by surgical resection. All patients were treated between 2015 and 2018 at the Department of Radiation Oncology, Charité University Hospital, Campus Virchow-Klinikum, Germany. In case of definitive CRT, radiotherapy treatment consisted of volumetric modulated arc or tomotherapy with an elective dose of 50.4 Gy and a consecutive boost to macroscopic tumor volumes with reduced margins of 0 to 9 Gy. Some patients received a simultaneous integrated boost to the metabolic tumor volume delineated on the pre-treatment FDG-PET up to 64 or 66 Gy. In the majority of patients concomitant chemotherapy consisted of weekly Carboplatin (AUC = 2.0) and Paclitaxel (50 mg/m^2^).

### PET imaging

Patients from Xiamen were scanned with a Discovery STE (General Electric Medical Systems, Milwaukee, WI, USA). Data acquisition started 67 ± 22 min (range 50–140 min) after injection of 142–548 MBq FDG (3D PET acquisition, 90 s acquisition time per bed position). PET data were reconstructed using CT-based attenuation-weighted OSEM reconstruction (2 iterations, 20 subsets, 6 mm FWHM Gaussian filter). Voxel size was 4 × 4 × 5 mm.

Patients from Berlin were scanned with a Gemini TF 16 Astonish (Philips Medical Systems, Cleveland, OH, USA). Data acquisition started 71 ± 9 min (range 60–86 min) after injection of 236–248 MBq FDG (3D PET acquisition, 90 s acquisition time per bed position). PET data were reconstructed using BLOB-OS-TF reconstruction (Philips Astonish TF technology: 3 iterations, 33 subsets). Voxel size was 2 × 2 × 2 mm and was re-scaled to 4 × 4 × 5 mm.

### Image analysis

For the radiomics analysis the metabolically active part of the primary tumor was delineated in the PET data by an automatic algorithm based on adaptive thresholding that considers the local background^16,17^. The resulting delineation was inspected visually by an experienced observer and, if necessary, manually corrected. In case of neighboring affected lymph nodes or high physiological tracer uptake (e.g. within the myocardium) these regions were manually subtracted. Tumor delineation was performed with the ROVER software, version 3.0.34 (ABX GmbH, Radeberg, Germany). Subsequently the DICOM data containing the structure sets of the tumors were exported and imported in 3D Slicer (https://www.slicer.org, version 4.8.1). Extraction of radiomics parameters was performed in all PET scans using the plugins SlicerRadiomics (Revision 8e5f1e8).

SlicerRadiomics based on PyRadiomics (version 2.0.1) is an open-source python package for the extraction of radiomics features from medical images^31^. We extracted seven classes of radiomics features with a total of 105 features according to the recommendations of Reuzé and colleagues dedicated to radiomics of PET images^32^. Shape (13 features), first order statistics (18 features), gray level co-occurrence matrix (glcm, 23 features), gray level size zone matrix (glszm,16 features), gray level run length matrix (glrlm,16 features), neighboring gray tone difference matrix (ngtdm, 5 features) and gray level dependence matrix (gldm,14 features). For a detailed list see Supplementary Table S4. All 105 radiomics features were calculated on the PET image data sets on a specified voxel-size of 4X4X5 millimeters. Suplementary Table S5 shows the procedure of image analysis according to IBSI recording guidelines^33^. Absolute gray-level discretization of PET images was performed using the fixed bin width approach and a bin size of 25, the rationale for using this approach was one publication showing a potential superiority of this approach compared to lesion-relative resampling of FDG-PET images^34^. Texture matrices were calculated as mean of texture values obtained from each normalized matrix in each direction with distance of 1 pixel/voxel. Importantly, some aspects of the PyRadiomics approach differ from ISBI guidelines, a detailed explanation can be found within the PyRadiomics documentation (https://pyradiomics.readthedocs.io/en/latest/faq.html). Auto-evaluation acording to the radiomics quality score was performed and revealed a score of 44% (Supplementary Table S7)^35^.

### Statistical analysis

First feature selection was performed in the training cohort of 100 patients. Primary endpoint was DFS, which was defined as the time between the first fraction of radiotherapy and any loco-regional tumor recurrence, distant metastasis or death of any cause. Secondary primary tumors were not regarded as an event. Further additional endpoints were overall survival (OS), local control (LC) and freedom from distant metastases (FFDM). It was pre-specified to select three or four radiomics features for the generation of a radiomics signature to avoid statistical overfitting and excessive selection of parameters potentially inter-correlated to each other. Radiomics signatures were calculated for each endpoint separately, i.e. each signature contains different parameters and different weighting. LASSO-COX regression was repeated 100 times in R (Comprehensive R Archive Network, https://www.r-project.org, version 3.4.1). Up to four radiomics parameters that were most frequently selected as significant (p < 0.05) by repeated Lasso-Cox regression analyses were implemented into the radiomics signature. The active coefficient for all features that showed significant association with outcome upon Lasso-Cox analyses were calculated for each run separately. The radiomics signature is a linear combination of the Lasso-Cox-Coefficient mean of all 100 runs and the patient specific value of the selected radiomics features. For better interpretability of the radiomics signatures we multiplied each radiomics signature by ten to avoid very small decimal values. The radiomics signatures for each endpoint were calculated independently and were tested for significance using the log-rank test. Additionally for dichotomization and generation of cuttoff values Kaplan–Meier curves were visually inspected using the software X-Tile (version 3.6.1) ^36^. The clinical rational for this study was pre-therapeutic detection of patients who are resistant to CRT and do not benefit from this treatment approach. Therefore cutoff values were selected in a way to detect a small proportion of patients (10–20%) with a dismal outcome applying current CRT based standard approaches. Univariate analysis of clinical parameters and radiomics signatures was performed for all endpoints. Supplementary Fig. S2 illustrates two high-risk patients with the same radiomics signature. To ensure independence of known prognostic factors, parameters with at least a trend for significance (p ≤ 0.1) in univariate analysis were tested by multivariate cox-regression analyses using SPSS (IBM, Armonk, New York, version 24).

For validation of the model, PET feature extraction and calculation of the radiomics signatures were performed in the same way as in the internal validation cohort using the coefficients of the internal validation cohort. After successful internal validation of cutoff values for the respective radiomics signatures, external validation was performed in the same manner.

### Ethical approval

The studies were approved by the Institutional Review Boards of the participating centers (Ethics Committee of the first affiliated hospital of Xiamen University and Charité's Ethics Committee) and the joint-analysis was additionally approved by the Institutional Review Board of the last authors’s institution (Charité's Ethics Committee, application number EA2/122/17) and was conducted in accordance with the guidelines of the International Conference on Harmonization/Good Clinical Practice and the principles of the Declaration of Helsinki. All patients provided written informed consent that their pseudonymized data can be used for scientific purposes and publications.

## Supplementary information

Supplementary Information.

## Data Availability

The data to generate the Radiomics signatures generated during this study are included in this published article (and its Supplementary Information files). The datasets analysed during the current study are not publicly available due to data safety policies but are available from the corresponding author on reasonable request.
