# Supplementary material for: A FDG-PET radiomics signature detects esophageal squamous cell carcinoma patients who do not benefit from chemoradiation
Source: Sci Rep. 2020 Oct 19;10:17671. doi: 10.1038/s41598-020-74701-w (PMC7573602; doi:10.1038/s41598-020-74701-w)

**A FDG-PET radiomics signature detects esophageal squamous cell carcinoma patients who do not benefit from chemoradiation**

Yimin Li^1,2#^ & Marcus Beck^3#^, Tom Päßler^3^, Chen Lili^1^, Wu Hua^4^, Ha Dong Mai^3^, Holger Amthauer^5^ Matthias Biebl^6^, Peter C Thuss-Patience^7^, Jasmin Berger^3^, Carmen Stromberger^3^, Ingeborg Tinhofer^3,8^, Jochen Kruppa^9^, Volker Budach^3^, Frank Hofheinz^10^, Quin Lin^1^*^,#^ & Sebastian Zschaeck^3,11#^

**Supplementary Information**

**Supplementary Table 1**: Patient and treatment characteristics

| **Training cohort (n= 100)** | |
| --- | --- |
| Tumor grading | |
| x | 9 (9%) |
| 1 | 10 (10)% |
| 2 | 62 (62%) |
| 3 | 16 (16%) |
| 4 | 3 (3%) |
| T stage | |
| 1 | 2 (2%) |
| 2 | 7 (7%) |
| 3 | 22 (22%) |
| 4 | 69 (69%) |
| N stage | |
| 0 | 36 (36%) |
| 1 | 36 (36%) |
| 2 | 27 (27%) |
| 3 | 1 (1%) |
| UICC stage |  |
| I | 1 (1%) |
| II | 17 (17%) |
| III | 13 (13%) |
| IVA | 69 (69%) |
| Age | Average: 63 (42 – 90) years |
| Gender | 76 male (76 %) |
| Chemotherapy | |
| Platinum + 5-fluorouracil | 27 (27%) |
| Platinum + taxane | 36 (36%) |
| Prescribed radiation dose | 50 – 66 Gray |
| **Internal validation cohort (n = 52)** | |
| Tumor grading | |
| x | 5 (9.6%) |
| 1 | 6 (11.5%) |
| 2 | 34 (65.4%) |
| 3 | 6 (11.5%) |
| 4 | 1 (1.9%) |
| T stage |  |
| 1 | 2 (3.8%) |
| 2 | 2 (3.8%) |
| 3 | 19 (36.5%) |
| 4 | 28 (53.8%) |
| N stage |  |
| 0 | 17 (32.7%) |
| 1 | 20 (38.5%) |
| 2 | 13 (25%) |
| 3 | 1 (1.9%) |
| UICC stage |  |
| I | 1 (1.9%) |
| II | 9 (17.3%) |
| III | 14 (26.9%) |
| IVA | 28 (53.8%) |
| Age | Average: 62 (42-87) years |
| Gender | 41 male (78.8%) |
| Chemotherapy |  |
| Platinum + 5-fluorouracil | 15 (28.8%) |
| Platinum + taxane | 15 (28.8%) |
| Prescribed radiation dose | 50 - 64 Gray |
| **External validation cohort (n = 32)** | |
| Tumor grading |  |
| x | 4 (13%) |
| 1 | - |
| 2 | 20 (62%) |
| 3 | 8 (25%) |
| T stage |  |
| 0 or X | 5 (16%) |
| 1 | - |
| 2 | 8 (25%) |
| 3 | 15 (47%) |
| 4 | 4 (12%) |
| N stage |  |
| X | 2 (6%) |
| 0 | 5 (16%) |
| 1 | 14 (44%) |
| 2 | 9 (28%) |
| 3 | 2 (6%) |
| UICC stage |  |
| II | 13 (41%) |
| III | 12 (37%) |
| IV | 7 (22%) |
| Age | Average: 64 (36-88) years |
| Gender | 17 (53%) male |
| Chemotherapy |  |
| Platinum + 5-fluorouracil | 3 (9%) |
| Platinum + taxane | 24 (75%) |
| Other | 5 (16%) |
| Prescribed radiation dose | 41.4 – 66 Gray |

**Supplementary table 2**: Selected parameters and their weighting of the radiomics signatures for all investigated endpoints (DFS, OS, LC).

| **DFS signature** | | **OS signature** | | **LC signature** | |
| --- | --- | --- | --- | --- | --- |
| Features | Weitghting | Features | Weitghting | Features | Weitghting |
| Shape_Minor Axis | 0.01063645 | Shape_Minor Axis | 0.00369795 | shape_Maximum2DDiameterSlice | 0.00915825 |
| glcm_Idn | 6.81604077 | glcm_Idn | 9.54091234 | shape_Major Axis | 0.00247585 |
| Firstorder_10 Percentile | 1.073e-05 | Firstorder_10 Percentile | 4.7301e-06 | shape_Surface Area | 0.00002850 |
|  |  | glcm_JointEntropy | 0.01161578 | shape_Maximum2DDiameterColumn | 0.00768968 |

**Supplementary table 3**: Correlation between respective radiomics signatures and clinical/ treatment parameters (training cohort, Spearman-correlation).

|  | **Radiomics Signature for DFS** | **Radiomics signature for LC** | **Radiomics Signature for OS** |
| --- | --- | --- | --- |
| **Type of chemotherapy** | r = 0.13  p = 0.21 | r = 0.162  p = 0.108 | r = 0.171  p = 0.089 |
| **UICC stage** | r = 0.071  p = 0.481 | r = -0.17  p = 0.87 | r = 0.044  p = 0.667 |
| **Tumor grading** | r = -0.053  p = 0.617 | r = -0.083  p = 0.433 | r = -0.089  p = 0.402 |
| **Gender** | r = -0.071  p = 0.483 | r = 0.064  p = 0.526 | r = 0.022  p = 0.826 |

**Supplementary table 4**: List of extracted radiomics features with corresponding abbreviations.

| Feature Class | Features |
| --- | --- |
| Shape | Mesh Volume, Surface Area,  Surface Area to Volume ratio, Sphericity, Maximum 3D diameter, Maximum 2D diameter (Slice), Maximum 2D diameter (Column), Maximum 2D diameter (Row), Major Axis Length, Minor Axis Length, Least Axis Length, Elongation, Flatness |
| First Order Features | Energy, Total Energy, Entropy, Minimum,  10th percentile, 90^th^ percentile, Maximum, Mean, Median, Interquartile Range, Range, Mean Absolute Deviation (MAD), Robust Mean Absolute Deviation (rMAD), Root Mean Squared (RMS), Skewness, Kurtosis, Variance,Uniformity |
| Gray Level Co-occurrence Matrix (glcm) | Autocorrelation, Joint Average, Cluster Prominence, Cluster Shade, Cluster Tendency, Contrast, Correlation, Difference Average, Difference Entropy, Difference Variance, Joint Energy, Joint Entropy, Informational Measure of Correlation (IMC) 1, Informational Measure of Correlation (IMC) 2, Inverse Difference Moment (IDM), Inverse Difference Moment Normalized (IDMN), Inverse Difference (ID), Inverse Difference Normalized (IDN), Inverse Variance, Maximum Probability, Sum Average, Sum Entropy, Sum of Squares |
| Gray Level Size Zone Matrix (glszm) | Small Area Emphasis (SAE), Large Area Emphasis (LAE), Gray Level Non-Uniformity (GLN), Gray Level Non-Uniformity Normalized (GLNN), Size-Zone Non-Uniformity (SZN), Size-Zone Non-Uniformity Normalized (SZNN), Zone Percentage (ZP), Gray Level Variance (GLV), **Zone Variance (ZV),** Zone Entropy (ZE),  Low Gray Level Zone Emphasis (LGLZE),  High Gray Level Zone Emphasis (HGLZE),  Small Area Low Gray Level Emphasis (SALGLE), Small Area High Gray Level Emphasis (SAHGLE), Large Area Low Gray Level Emphasis (LALGLE), Large Area High Gray Level Emphasis (LAHGLE) |
| Gray Level Run Length Matrix (glrlm) | Short Run Emphasis (SRE), Long Run Emphasis (LRE), Gray Level Non-Uniformity (GLN), Gray Level Non-Uniformity Normalized (GLNN), Run Length Non-Uniformity (RLN), Run Length Non-Uniformity Normalized (RLNN), Run Percentage (RP), Gray Level Variance (GLV), Run Variance (RV), Run Entropy (RE), Low Gray Level Run Emphasis (LGLRE), High Gray Level Run Emphasis (HGLRE), Short Run Low Gray Level Emphasis (SRLGLE), Short Run High Gray Level Emphasis (SRHGLE), Long Run Low Gray Level Emphasis (LRLGLE), Long Run High Gray Level Emphasis (LRHGLE) |
| Neighboring Gray Tone Difference Matrix (ngtdm) | Coarseness, Contrast, Busyness, Complexity, Strength |
| Gray Level Dependence Matrix (gldm) | Small Dependence Emphasis (SDE), Large Dependence Emphasis (LDE), Gray Level Non-Uniformity (GLN), Dependence Non-Uniformity (DN), Dependence Non-Uniformity Normalized (DNN), Gray Level Variance (GLV), Dependence Variance (DV), Dependence Entropy (DE), Low Gray Level Emphasis (LGLE), High Gray Level Emphasis (HGLE), Small Dependence Low Gray Level Emphasis (SDLGLE), Small Dependence High Gray Level Emphasis (SDHGLE), Large Dependence Low Gray Level Emphasis (LDLGLE), Large Dependence High Gray Level Emphasis (LDHGLE) |

**Supplementary table 5:** Adherence to IBSI Reporting guidelines.

| **General** | |
| --- | --- |
| Image acquisition | Xiamen: PET-CT scanner Discovery STE (General Electric Medical Systems, Milwaukee, WI, USA), Data acquisition started 67±22 min (range 50–140 min) after injection of 142–548 MBq FDG (3D PET acquisition, 90 s acquisition time per bed position).  Berlin: PET-CT scanner Gemini TF 16 Astonish (Philips Medical Systems, Cleveland, OH, USA). Data acquisition started 71±9 min (range: 60–86 min) after injection of 236–248 MBq FDG (3D PET acquisition, 90 s acquisition time per bed position) |
| Volumetric analysis | Volumes were analyzed as fully-connected 3D volumes |
| Workflow structure | Re-Scaling of images, (semi-) atomatic delineation and export of regions of interest and dicom data with ROVER. Analysis of exported images with 3D slicer using the SlicerRadiomics plugin |
| Software | ROVER, version 3.0.34 (ABX GmbH, Radeberg, Germany).  3D Slicer, version 4.8.1 (<https://www.slicer.org>)  SlicerRadiomics plugin (Revision 8e5f1e8) |
| **Image preprocessing** | |
| Conversion | Xiamen: PET data were reconstructed using CT-based attenuation-weighted OSEM reconstruction (2 iterations, 20 subsets, 6 mm FWHM Gaussian filter)  Berlin: PET data were reconstructed using BLOB-OS-TF reconstruction (Philips Astonish TF technology: 3 iterations, 33 subsets) |
| Processing | Re-scaling to uniform voxel sizes of 4X4X5 mm |
| **ROI segmentation** | Semi-automatic delineation of primary tumor volumes using the algorithm implemented in ROVER. |
| **Interpolation** | |
| Voxel dimensions | original voxel dimensions between 2X2X2 mm and 4X4X5 mm, interpolation of all voxels to 4X4X5 mm. |
| Image interpolation method | Trilinear interpolation |
| Intensity rounding | Not applicable |
| ROI interpolation method | No additional interpolation of ROIs was performed |
| ROI partial volume | Not applicable (usually relatively large primary tumor volumes) |
| **ROI resegmentation** | Re-segmentation was only performed in few cases as described in Methods. Especially in case of proximity to involved lymphnodes. |
| **Image discretization** | |
| Discretization method | SUV discretization using a fixed intensity resolution of SUV units |
| Discretization parameters | fixed bin width of 25 |
| **Feature calculation** | |
| Feature set | See supplementary table 2 |
| Feature parameters | All parameters according to the SlicerRadiomics plugin (see Methods).  No merging by slice or of directional texture matrices was performed. |
| **Calibration** | |
| Image-processing steps | All image-processing steps match IBSI benchmarks, except for differences in gray value discretization and resampling (see <https://pyradiomics.readthedocs.io/en/latest/faq.html> for details) |
| Feature calculation | Feature calculation match IBSI benchmarks |

| Parameter | Univariate variance analysis between training cohort and internal validation cohort | Univariate variance analysis between training cohort, internal validation cohort and external validation cohort |
| --- | --- | --- |
| Shape_Minor Axis | p = 0.28 | p = 0.56 |
| glcm_Idn | p = 0.56 | **p < 0.001** |
| Firstorder_10 Percentile | p = 0.17 | **p < 0.001** |
| SUV_max_ | p = 0.29 | **p < 0.001** |

**Supplementary Table 6**: Univariate variance analyses of included radiomic parameters between training cohort and internal validation cohort and all three cohorts.

**Supplementary Table 7**: Radiomics quality score evaluation

| **Criteria** | **Points** |
| --- | --- |
| Image protocol quality | 1 |
| Segmentation quality | 1 |
| phantom study | 0 |
| Imaging at multiple time points | 0 |
| Feature reduction | 2 |
| Multivariable analysis with non radiomic features | 1 |
| Detect and discuss biological correlates | 0 |
| Cut-off analyses | 1 |
| Discrimination statistics | 0 |
| Calibration statistics | 0 |
| Prospective study | 0 |
| Validation | 5 |
| Comparison to gold standard | 2 |
| Potential clinical utility | 2 |
| Cost-effectiveness analysis | 0 |
| Open science and data | 1 |
| Total score | 16/ 36 points = 44% |

**Supplementary figure 1:** Training cohort. Kaplan-Meier estimates with prognostic groups split by metabolic tumor volume (MTV) into high and low-risk population. Investigated endpoint: DFS.

**Supplementary figure 2:** Training cohort. Kaplan-Meier estimates with prognostic groups split by metabolic tumor volume (MTV) into high and low-risk population. Investigated endpoint: OS.

**Supplementary figure 3:** Training cohort. Kaplan-Meier estimates with prognostic groups split by metabolic tumor volume (MTV) into high and low-risk population. Investigated endpoint: LC.

**Supplementary figure 4:** Training cohort. Kaplan-Meier estimates with prognostic groups split by maximum standardized uptake value (SUVmax) into high and low-risk population. Investigated endpoint: DFS.

**Supplementary figure 5:** Training cohort. Kaplan-Meier estimates with prognostic groups split by maximum standardized uptake value (SUVmax) into high and low-risk population. Investigated endpoint: OS.


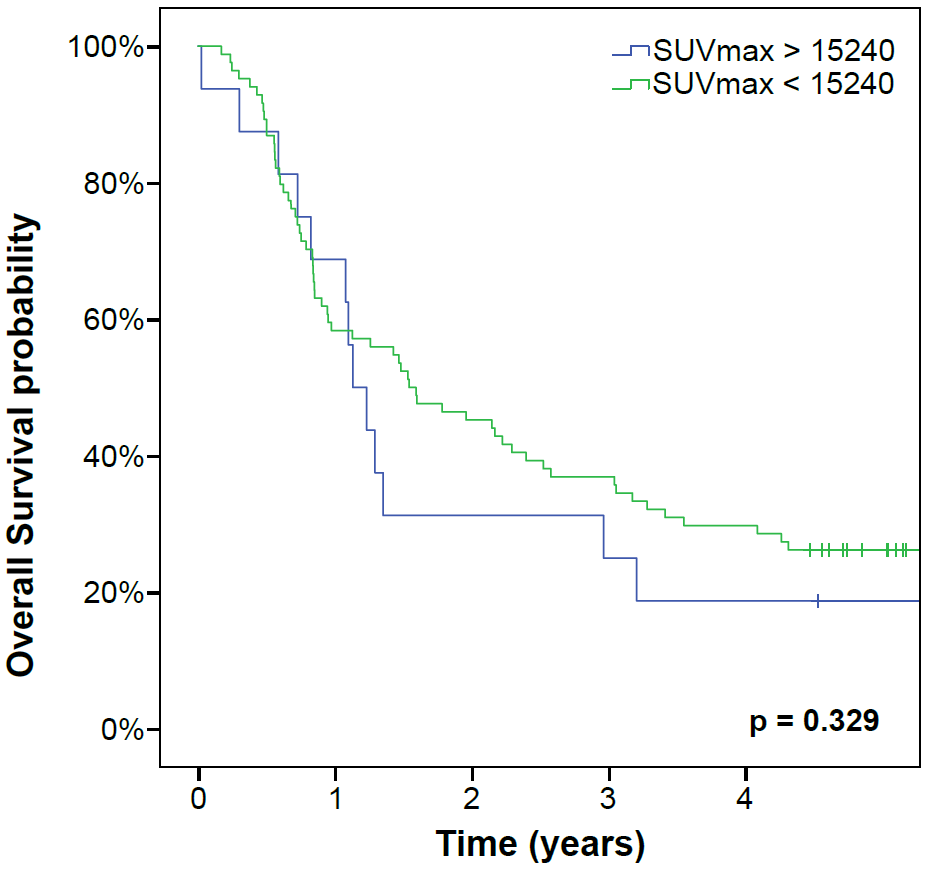


**Supplementary figure 6:** Training cohort. Kaplan-Meier estimates with prognostic groups split by maximum standardized uptake value (SUVmax) into high and low-risk population. Investigated endpoint: LC.

**Supplementary figure 7:** Internal validation cohort. Kaplan-Meier estimates with prognostic groups split by metabolic tumor volume (MTV) into high and low-risk population. Investigated endpoint: LC. Cutoff value according to training cohort.

**Supplementary figure 8:** Internal validation cohort. Kaplan-Meier estimates with prognostic groups split by radiomic scores into high and low-risk population. Investigated endpoint: LC. Cutoff value according to training cohort. Data was augmented based on the training dataset distribution using Synthetic Minority Oversampling Technique (SMOTE).

**Supplementary figure 9:** External validation cohort. Kaplan-Meier estimates with prognostic groups split by metabolic tumor volume (MTV) into high and low-risk population. Investigated endpoint: LC. Cutoff value according to training cohort.

**Supplementary figure 10:** Example of two high risk patients and the respective atomatic delineations for the primary tumor. Both patients had a DFS Radiomic signature score of 6.9, while the metabolic tumor volume of the patient shown above is 35ml, the metabolic tumor volume of the patient below is only 16ml.


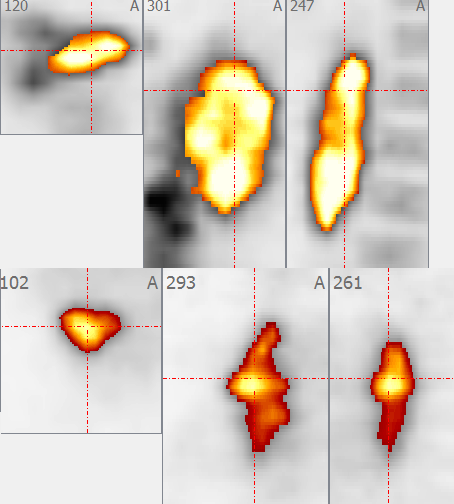

Supplement: Supplementary file 1 — Supplementary Information. [file 41598_2020_74701_MOESM1_ESM.docx]
